# Microbiological profile of corneal ulcer cases diagnosed in a tertiary care ophthalmological institute in Nepal

**DOI:** 10.1186/s12886-016-0388-9

**Published:** 2016-11-29

**Authors:** Sharmila Suwal, Dinesh Bhandari, Pratigya Thapa, Mohan Krishna Shrestha, Jyoti Amatya

**Affiliations:** 1 Department of Microbiology, Trichandra Multiple College, Ghantaghar, Kathmandu, Nepal; 2 Public Health Research Laboratory, Institute of Medicine, Tribhuvan University Teaching Hospital, Maharajgunj, Kathmandu, Nepal; 3 Tilganga Institute of Ophthalmology, Kathmandu, Nepal

**Keywords:** Corneal ulcer, Microbiological profile, Nepal

## Abstract

**Background:**

Corneal ulcer, a major cause of monocular blindness in developing countries has consistently been listed as the major cause of blindness and visual disability in many of the developing nations in Asia, Africa and the Middle East, ranking second only to cataract. This study was carried out to determine the microbiological profile of corneal ulcer cases diagnosed among patients visiting Tilganga Institute of Ophthalmology (TIO), Nepal.

**Methods:**

A total of 101 corneal scrapping samples were tested for routine culture and antibiotic susceptibility at the pathology department of TIO Nepal from April to October 2014. Microorganisms were identified by using standard microbiological procedures following the manual of American Society for Microbiology (ASM) and their antibiotic susceptibility test, performed by Kirby-Bauer disc diffusion method in conformity with the CLSI guideline.

**Results:**

Out of 101 samples analyzed, 44.6% (45/101) showed positive growth with bacterial isolates i.e., 56% (25/45), more prevalent than fungus i.e., 44% (20/45). Among bacteria *Streptococcus pneumoniae* (31.1%, *N* = 14) was isolated in highest number whereas *Fusarium* (13.4%, *N* = 6) was the most common fungus species. *Pseudomonas aeruginosa* was the only Gram negative bacteria isolated from corneal ulcer cases. All bacterial isolates were found to be susceptible to the quinolone group of antibiotics (moxifloxacin followed by ofloxacin and ciprofloxacin).

**Conclusions:**

These findings showcase the current trend in the microbiological etiology of corneal ulcer in Nepal, which have important public health implications for the treatment as well as prevention of corneal ulceration in the developing world.

## Background

Corneal ulcer, an inflammatory or more seriously, infective condition of the cornea involving disruption of its epithelial layer with involvement of the corneal stroma, is one of the major causes of monocular blindness after unoperated cataract in many of the developing nations in Asia, Africa and the Middle East. [1, 2] It is a sight threatening disorder that affects both males and females across all age groups worldwide. In the United States alone, 930,000 cases seek outdoor medical attention and 58,000 cases visit the emergency department [3]. The annual financial burden borne in United States in direct health care expenditures due to cases related to corneal ulcer and keratitis is estimated to be $175 million [3]. In the developing countries, the financial burden related to this diesease is undetermined but speculated to be calamitous [4].

Herpes Simplex Virus type 1 (HSV-1) is the most common cause of corneal ulcer but other etiological agents frequently associated with corneal ulcer include bacteria (*Staphylococcus aureus*, *Staphylococcus epidermidis*, *Streptococcus pneumoniae*, *Streptococcus pyogenes*, *Moraxella species*, *Pseudomonas aeruginosa*, *Proteus species*, *Klebsiella pneumoniae*, *Yersinia* species and *Escherichia coli*), fungus (*Candida albicans*, *Aspergillus flavus*, *Fusarium solani*, *Penicilium* species and *Aspergillus fumigates*) and parasites (*Acanthamoeba*) [5–8]. In addition, *Pseudomonas* a Gram negative opportunistic bacteria is also commonly associated with keratitis arising from contact lens wear, which ultimately leads to corneal ulcer [9]. The etiology of corneal ulcer varies disproportionately in different geographical regions with highest proportion of bacterial corneal ulcers reported from North America, Australia, Netherlands, and Singapore and that of fungal corneal ulcer from India and Nepal [10].

Corneal ulcer is an ophthalmic condition requiring prompt medical attention. Thus precise knowledge of the causative agents and their susceptibility patterns is important for deciding the proper course of treatment. To the best of our knowledge, the microbial etiology of corneal ulcer and its management in Nepal has remained unclear [11–13]. Thus, the aim of this research is to analyze the etiology of corneal ulcer in Nepal and to determine the antibiotic susceptibility pattern of bacterial isolates thereby reducing antibiotic misuse and the incidence of microbial drug resistance.

## Methods

### Study setting, design and study population

This hospital based descriptive cross-sectional study was carried out between April-October 2014 at the Pathology Laboratory of Tilganga Institute of Ophthalmology (TIO), Nepal, which is the largest community-based non-governmental organization committed to providing quality ophthalmic care in Nepal. Corneal scrapings received for culture from the corneal ulcer suspected patients of all age groups as requested by ophthalmologists were included in the study and patients with perforated corneal ulcer were excluded. Since, no pre-defined sample size was set prior to the inception of the study; 101 corneal scarping samples received during the period of 7 months were included in the study.

### Sample collection and laboratory processing

Corneal scrapings from both the leading edge as well as base of each ulcer were collected under aseptic conditions by ophthalmologists under the magnification of a slit lamp after instillation of 4% Xylocaine, using a flame sterilized Kimura spatula. Samples thus obtained were then processed by standard operating procedure following the manual of American Society for Microbiology [14]. Briefly, the samples were inoculated in routine culture media (Blood agar, Chocolate agar and Sabouraud dextrose agar) [Hi media Laboratory Ltd, Mumbai, India] and subjected for microscopic examination as KOH wet mount. Likewise, Lacto phenol cotton blue/Gram’s stain was prepared for morphology based identification of the fungus and bacteria and cultural characteristics and biochemical properties were determined in compliance with ASM manual [14]. Acid-Fast Staining (Modified Kinyoun) was performed in order to confirm *Nocardia* species [15].

### Antibiotic susceptibility test

Antibiotic susceptibility of the bacterial isolates was performed using a modified Kirby- Bauer disc diffusion method and the results were interpreted according to the CLSI guideline [16]. The antibiotic discs used were amikacin (30μg), chloramphenicol (30μg), ciprofloxacin (5μg), ofloxacin (5μg), moxifloxacin (5μg), ceftazidime (30μg), tetracycline (30μg) and azithromycin (15μg) (Hi Media Laboratory Ltd, Mumbai, India).

### Data management and analysis

The data obtained was entered in Microsoft Office Excel 2007 and analyzed by Statistical Package for Social Sciences (SPSS) version 16.0. Frequency and percentages were calculated and two-tailed Pearson’s Chi-square test was used to test the significance of attributes between study variables. The *p*-value < 0.05 was considered statistically significant.

## Results

Of the 101 samples investigated, 44.6% (45/101) were positive for etiology in both microscopy and culture, indicating that smear microscopy was highly predictive of culture positivity. Among the 45 (44.6%) positive samples, bacterial isolates were recovered in 56% (25/45) and fungal isolates in 44% (20/45) of the cases. *S. pneumoniae* 31.1% (14/45) was the most commonly isolated bacteria followed by viridians group streptococci. *Nocardia* species and *Bacillus* species 6.7% (3/45) was also detected. *Fusarium* species 13.4% (6/45) were the most commonly isolated fungus followed by *Aspergillus flavus* and unidentified dematiaceous fungus 11.1% (5/45), *Curvularia* 4.4% (2/45), *Bipolaris* species and *Exserohilum* species 2.2% (1/45) (Table 1).

**Table 1:** Etiology of Corneal ulcers

	Etiologies	Frequency (%)
Bacterial corneal ulcer (*N* = 25)	*Bacillus* species	3 (6.7)
	*Nocardia* species	3 (6.7)
	*Pseudomonas aeruginosa*	1 (2.2)
	*Staphylococcus aureus*	1 (2.2)
	*Streptococcus pneumonia*	14 (31.1)
	Viridians group of streptococci	3 (6.7)
Fungal corneal ulcer (*N* = 20)	*Aspergillus flavus*	5 (11.1)
	*Bipolaris* species	1 (2.2)
	*Curvularia* species	2 (4.4)
	*Exserohilum* species	1 (2.2)
	*Fusarium* species	6 (13.4)
	Unidentified dematiaceous fungi	5 (11.1)
Total		45 (100)

### Gender and Agewise distribution of corneal ulcers suspected cases

There was a slight female dominance in the sex ratio (1.4:1) with females contributing 58% and males 42% among the total 45 positive samples (Table 2). The highest number of patients 40% (18/45) from positive case belonged to age group 51–60 (Table 2). There was no statistical signigifance (*p* > 0.05) between the gender or age of the cases and the incidence of corneal ulcer in this study.

**Table 2:** Demographic factors and clinical presentations of corneal ulcers

Demographic variables	Particulars (*N* = 101)	Corneal ulcer positive cases, *N* = 45 (%)^a^
Gender	Male (*n* = 53)	19 (42.2%)
	Female (*n* = 48)	26 (57.8%)
Age in years	<10 years (*n* = 4)	0
	11–20 years (*n* = 3)	1 (2.2%)
	21–30 years (*n* = 13)	4 (8.9%)
	31–40 years (*n* = 19)	8 (17.8%)
	41–50 years (*n* = 11)	3 (6.7%)
	51–60 years (n = 28)	18 (40%)
	61–70 years (*n* = 16)	7 (15.6%)
	71–80 years (*n* = 4)	2 (4.4%)
	>80 years (*n* = 3)	2 (4.4%)
Occupation	Agriculture (*n* = 59)	26 (57.8%)
	Others (*n* = 42)	19 (42.2%)
Education	Illiterate (*n* = 72)	33 (73.3%)
	Literate (*n* = 29)	12 (26.7%)
Trauma	Yes (*n* = 29)	13 (28.9%)
	No (*n* = 72)	32 (71.1%)

^a^The percentage has been derived, taking the total positive cases as denominator (*N* = 45)

### Socioeconomic factors and clincial presentation of corneal ulcers

Almost 57.8% of the culture positive cases were farmers and 73.3% of them were illiterate. Patient diagnosed via culture positivity for microbial etiology as the corneal ulcer cases presented with different clinical symptoms including ocular pain, redness of the eyes, decreased vision, white lesion and others (discharge, watering and foreign body sensation). Growth positivity for microbial etiology was statistically significant (*p* < 0.05) with trauma (28.9%) as an important clinical presentation among the positive cases (Table 2).

### Antibiotic susceptibility pattern of the bacterial isolates recovered

Among the eight different antibiotics used against the bacterial isolates, moxifloxacin showed 100% susceptibility followed by ofloxacin 92% and ciprofloxacin 88%. Both *S. pneumoniae* and viridians group of streptococci were 100% susceptible to all of the antibiotics used. *Nocardia* species were 66.67% resistant to azithromycin, ciprofloxacin and ofloxacin but 100% susceptible to amikacin, chloramphenicol and moxifloxacin. Although *P. aeruginosa* were sensitive to amikacin, ciprofloxacin, moxifloxacin and ofloxacin, they were resistant against ceftazidime, chloramphenicol and tetracycline (Table 3).

**Table 3:** Antibiotic susceptibility pattern of bacterial isolates

Organisms	Antibiotics Used	Susceptibility Patterns		Resistant
		Susceptible	Intermediate	
*Streptococcus pneumoniae* (*N* = 14)	Azithromycin	14	0	0
	Ceftazidime	14	0	0
	Chloramphenicol	14	0	0
	Ciprofloxacin	14	0	0
	Moxifloxacin	14	0	0
	Ofloxacin	14	0	0
Viridians group of streptococci (*N* = 3)	Azithromycin	3	0	0
	Ceftazidime	3	0	0
	Chloramphenicol	3	0	0
	Ciprofloxacin	3	0	0
	Moxifloxacin	3	0	0
	Ofloxacin	3	0	0
*Staphylococcus aureus* (*N* = 1)	Amikacin	1	0	0
	Ceftazidime	1	0	0
	Chloramphenicol	0	0	1
	Ciprofloxacin	0	1	0
	Moxifloxacin	1	0	0
	Ofloxacin	1	0	0
*Bacillus* species (*N* = 3)	Amikacin	3	0	0
	Azithromycin	1	0	2
	Chloramphenicol	1	0	2
	Ciprofloxacin	3	0	0
	Moxifloxacin	3	0	0
	Ofloxacin	3	0	0
*Nocardia* species (*N* = 3)	Amikacin	3	0	0
	Azithromycin	1	0	2
	Chloramphenicol	3	0	0
	Ciprofloxacin	1	0	2
	Moxifloxacin	3	0	0
	Ofloxacin	1	0	2
*Pseudomonas aeruginosa* (*N* = 1)	Amikacin	1	0	0
	Ceftazidime	0	0	1
	Chloramphenicol	0	0	1
	Ciprofloxacin	1	0	0
	Moxifloxacin	1	0	0
	Ofloxacin	1	0	0

## Discussion

Proper management and treatment of corneal ulcers, a major cause of blindness worldwide requires precise identification of the etiology so that an appropriate antimicrobial agent targeting the organism responsible can be administered on time. Nonetheless, the inconsistency in prevalence and causes of corneal blindness across geography and ethnic groups make it challenging to administer a standard set of protocols in order to lower the incidence of corneal ulcer [1]. Given these milieu, the awareness among ophthalmologists of regional epidemiological features, risk factors, and etiological data concerning this ophthalmic condition is necessary. Thus, we explored the etiological agent of corneal ulcer, identified associated risk factors and antibiotic susceptibility of bacterial isolates identified.

Although the culture positivity of 44.6% that we observed in Nepali populations is comparable to previous studies that reported 40–45%, culture positivity in this region [17, 18], we detected lower positivity than a previous study conducted at the same ophthalmic center [12]. The reason for such lower prevalence could be due to differences in methods used to ascertain positivity and difference in sample size. Alternatively, improved eye care services at ophthalmological facilities may have resulted in decreased incidence of corneal ulcer cases in Nepal.

The bacterial isolates accounted for 56% (25/45) and fungal isolates for 44% (20/45) of the total corneal ulcer cases which demonstrates the shift from fungi to bacteria as major agent associated with this disease in this region [10]. This transition from fungi to bacteria as major etiological agent in Kathmandu could be due to rapid urbanization and large reductions in agricultural practices within Kathmandu in the last few years (Table 2). Among the bacterial isolates *S. pneumoniae* 31.1% (14/45) showed higher prevalence which is in harmony with the findings of similar studies conducted elsewhere [12, 19]. *S. pneumoniae* is the major biological agent causing corneal ulcer in developing as well as industrial nations. The production of virulence factor pneumolysin favors *S. pneumonae* to establish infection in corneal epithelium [20]. Meanwhile, *Fusarium* species was the dominant fungi causing corneal ulcer which is in concordance with the finding of previous studies [18, 21, 22].

The infection ratio of male: female was found to be 0.7:1. This finding is not in conformity with several studies conducted elsewhere which have reported a higher susceptibility of male toward infection compared to female [7, 17, 18, 23]. The difference in ratio may be due to more exposure of female populaiton in agricultural and household activities in our context compared to those studies. However, the role of gender in corneal ulcer is always contradictory and further rigorous research is required. The highest number of patients, 40% (18/45) from corneal ulcer positive case belonged to age group 51–60. It is due to the fact that people of age between 51 and 60 years have many predisposing factors like CDK (climatic droplet keratopathy), dryness of the eyes, cataract surgery, glaucoma, macular degeneration, previous ocular surgeries and lid deformities due to trachomatous scarring which probably predispose this age group to corneal ulceration more than the other age groups [24]. However, in our study no statistical significance was established (*p* > 0.05) between the age of patient and corneal ulcer.

The higher prevalence of corneal ulcer was seen in the agricultural group (57.8%), which was similar to finding reported by Basak et al. [23]; but a marked contrast was seen with the study done in Ghana where only 16.1% corneal ulcer cases were associated with agricultural profession. This could be due to the differences in the occupational pattern between the two countires in consideration. However, no statistical significance (*p* > 0.05) was seen between the occupation and corneal ulcer in our case.

The age, gender, and education distributions of each cohort correspond to the population distributions of visual impairment as reported by the World Health Organization [25]. In this study, corneal ulcer was presented with higher prevalence among people receiving less education as has been the pattern reported by other researchers from around the globe [4, 23]. Individuals with lower education are ignorant and less conscious about their health. However, the culture positivity was not statistically significant (*p* > 0.05) with the education status of patients.

Ocular trauma or corneal injury has always been identified as a cause of corneal ulcer [8, 23]. In our study statistical significant (*p* < 0.05) was established between corneal ulcer and trauma (28.9%) as indicated by the culture positivity. Use of contact lenses has become one of the main reasons for microbial keratitis in the developed nations where they are broadly accessible, mainly in young adults [9, 26, 27]. In contrast to the reports cited above even a single case of corneal ulcer predisposed by contact lens wear was not reported. This may be because of the fact that contact lenses are, as yet not widely used in Nepal due to the extra financial burden borne on patient when opting to lenses instead of glasses/spectacles. Similarly, the less frequent isolation of *Pseudomonas* species may also be attributed to infrequent use of contact lens.

In the view of frequent reports of changing pattern of susceptibility among the bacteria, testing of clinical isolates for their susceptibility to antimicrobial drugs is necessary for selection of appropriate antibiotics or for changing an already administered drug. In this study, the isolated bacteria were tested against eight different antibiotics in the laboratory as recommended by CLSI [16]. Since, there are no susceptibility standards for topical antibiotic therapy in ophthalmology, the resistance determined in this study is based on the systemic susceptibility breakpoints. All the bacterial isolates (Gram positive and negative) were 100% susceptible to fourth generation quinolone antibiotic moxifloxacin, the drug of choice for bacteria incriminated with ophthalmic problems.

All the isolated *S. pneumoniae* and viridians group of streptococci were 100% susceptible to the entire panel of antibiotics used. Amikacin, ceftazidime, moxifloxacin and ofloxacin were found to be effective against *S. aureus. Nocardia* species were 66.67% resistant to ciprofloxacin, ofloxacin, azithromycin whereas, 100% susceptible to chloramphenicol, moxifloxacin, and amikacin. Similarly, *Bacillus* species were 66.67% resistant to chloramphenicol and azithromycin and 100% susceptible to amikacin, ciprofloxacin, moxifloxacin and ofloxacin. *P. aeruginosa* was resistant to chloramphenicol and ceftazidime and susceptible to aminoglycosides and quinolones. These results indicate that chloramphenicol should not be used routinely as the topical antibiotic of choice for corneal infection in Nepal, a view supported by studies in Australia, Singapore, and London [28].

However, failure to perform the susceptibility test of the antifungal agents against the fungal isolates comes under the short coming of this study. Had the resource limitation and financial constrains not restrained us from performing susceptibility test for fungal isolates, the findings generated would have been an updated guideline for Ophthalmologist in this region to choose an appropriate drug among the multiple empirical options available for treatment of corneal ulcer. An extensive microbiological study of corneal ulcer and keratitis with susceptibility testing of broad range of isolates recovered will be our future research preference.

## Conclusions

The findings of our study implicate use of moxifloxacin as the best therapeutic option in treatment of bacterial corneal ulcer cases and withdrawal of chloramphenicol from the treatment option due to its reduced susceptibility towards most of the causative agents (bacteria) of corneal ulcer isolated in our study. Early isolation of causative organism and treatment with intensive ocular antibiotics represent decisive steps in the management of corneal ulcer. Hence, a further study with larger sample size to look at the predictability of predisposing factors as well as the determination of susceptibility pattern of antifungal agents would be clinically valuable.

## Abbreviations

ASM: American Society of Microbiology
CDK: Climatic Droplet Keratopathy
CLSI: Clinical Laboratory Standard International
HSV-1: *Herpes Simplex Virus* type 1
SPSS: Statistical Package for Social Sciences
TIO: Tilganga Institute of Ophthalmology
